# Nicotinamide could reduce growth and cariogenic virulence of *Streptococcus mutans*

**DOI:** 10.1080/20002297.2022.2056291

**Published:** 2022-03-23

**Authors:** Yongwang Lin, Tao Gong, Qizhao Ma, Meiling Jing, Ting Zheng, Jiangchuan Yan, Jiamin Chen, Yangyang Pan, Qun Sun, Xuedong Zhou, Yuqing Li

**Affiliations:** a State Key Laboratory of Oral Diseases, National Clinical Research Center for Oral Diseases, Department of Operative Dentistry and Endodontics, West China Hospital of Stomatology, Sichuan University, Chengdu, Sichuan, China; b Key Laboratory of Bio-resources & Eco-environment of the Ministry of Education, College of Life Sciences, Sichuan University, Chengdu, Sichuan, China

**Keywords:** Nicotinamide, *Streptococcus mutans*, virulence, biofilm(s), caries

## Abstract

Dental caries is among the most prevalent chronic oral infectious diseases. *Streptococcus mutans*, a major cariogenic bacterial species, possesses several cariogenicity-associated characteristics, including exopolysaccharides (EPS) synthesis, biofilm formation, acidogenicity, and aciduricity. Nicotinamide (NAM), a form of vitamin B3, is a non-toxic, orally available, and inexpensive compound. The present study investigated the inhibitory effects of NAM on the cariogenic virulence factors of *S. mutans in vitro* and *in vivo*. NAM inhibited the growth of *S. mutans* UA159 and the clinical isolates. In addition, there was a decrease in the acid production and acid tolerance ability, as well as biofilm formation and EPS production of *S. mutans* after NAM treatment. Global gene expression profiling showed that 128 and 58 genes were significantly downregulated and upregulated, respectively, in NAM-treated *S. mutans* strains. The differentially expressed genes were mainly associated with carbohydrate transport and metabolism, glycolysis, acid tolerance. Moreover, in a rat caries model, NAM significantly reduced the incidence and severity of smooth and sulcal-surface caries *in vivo*. NAM exhibited good antimicrobial properties against *S. mutans*, indicating its potential value for antibiofilm and anti-caries applications.

## Introduction

Dental caries constitutes a prevalent public health issue worldwide and can damage human health, affect the quality of life, and increase the financial burden [1]. *Streptococcus mutans* has been considered a major cariogenic bacterial species [2]. The characteristics making *S. mutans* an efficient cariogenic pathogen within the oral microbiota include production of exopolysaccharides (EPS) and acids, living in a biofilm lifestyle, and mounting stress resistance [3]. EPS production, especially water-insoluble glucans, serve as the key virulence factor of *S. mutans* [4]. *S. mutans* synthesizes glucans via glucosyltransferases (GTFs) [5]. The glucans provide binding sites for bacteria and serve as the main component of the biofilm matrix, which can accommodate and protect various bacteria and maintain a local low-pH environment [3,6]. Once the acidic environment has been established and sustained, the commensals might vanish, and *S. mutans* and other potent acidogenic/aciduric organisms flourish to become dominant and promote carious lesions [7,8]. Hence, suppressing cariogenic virulence factors of *S. mutans* could be an appealing approach to prevent dental caries.

Despite significant advances in *S. mutans* treatment research, there are still many hurdles. Fluoride and chlorhexidine (CHX) are two widely used anti-caries agents and have shown inhibitory effects on *S. mutans*. However, fluoride exhibits toxic effects such as fluorosis when used in high concentrations, and CHX is not suitable for daily use due to its adverse effects, including calculus formation and tooth staining [9]. Concerning other strategies, EPS hydrolases can digest *S. mutans* biofilm EPS matrix, but they have no antibacterial activity and have a high cost [10]. Nanomaterials have been tested to inhibit *S. mutans* virulence factors [11], and gene-editing tools can target *S. mutans* virulence genes and regulatory systems; however, the proper application form and safety still require further research [12]. Given the above, searching for a safe and effective strategy to inhibit *S. mutans* cariogenic characteristics is necessary.

Nicotinamide (NAM), an amide form of vitamin B3, is a safe agent and can be used in high doses for various therapeutic applications [13]. Several studies have assured the safety of NAM with the currently widely used long-term dietary intake of 500–1,000 mg/day. NAM has come into medical use since 1937 as a ‘pellagra-preventing’ agent and is prescribed topically to treat several inflammatory skin conditions, including acne vulgaris and atopic dermatitis [14]. Moreover, nicotinamide exhibits cytoprotective qualities in immune system dysfunction, aging-associated conditions, and diabetes [15]. In recent years, the antimicrobial activity of NAM has been established against several microorganisms, including *Mycobacterium tuberculosis, Staphylococcus aureus, Candida albicans*, and African trypanosomes [16–19], but the effects of NAM on cariogenic bacteria have not been reported yet.

This study investigated the effect of NAM on growth, acid production, acid resistance, biofilm formation, and EPS synthesis of *S. mutans*. The underlying function mechanism of NAM was explored based on RNA-sequencing analysis. Moreover, we assessed the effect of NAM on *S. mutans* cariogenicity in a rat caries model.

## Materials and methods

### Test bacteria and growth conditions

The test bacteria, *S. mutans* UA159 was provided by the State Key Laboratory of Oral Diseases, Sichuan University. *S. mutans* clinical strains (Table S1) were isolated from the oral cavity of pediatric patients in the West China Hospital of Stomatology with the approval by the International Ethical Committee of the West China School of Stomatology, Sichuan University (WCHSIRB-D-2015–084) [20]. All the bacteria were grown routinely in brain heart infusion (BHI) broth (BD, Sparks) at 37°C under 5% CO_2_. For biofilm growth, 1% sucrose (Sigma) was added to the BHI broth (designated BHIS) [21].

### Specimen preparation

Nicotinamide (NAM) powder purchased from Solarbio (China?) was dissolved in double-distilled water to prepare a stock solution with a concentration of 512 μg/μL and then diluted by BHI (for planktonic bacteria cultivation) and BHIS (for biofilm cultivation). The minimal inhibitory concentrations (MICs) of NAM to *S. mutans* were examined by the microdilution method. The MIC value was 32 μg/μL (Table S2), and we determined the working concentrations of NAM as 4, 8, and 16 μg/μL in the *in vitro* tests.

### Planktonic growth assays

For the planktonic growth assays, overnight cultures of UA159 and clinical *mutans* strains were subcultured in BHI until the OD_600nm_ = 0.5 was achieved and inoculated at a dilution of 1:100 into fresh BHI broth containing various concentrations of the compound nicotinamide (4, 8 and 16 μg/μL). BHI without NAM served as the control group, followed by incubation at 37°C for 24 h in 96-wells. The wells without bacteria received the media, only serving as blanks. Bacterial growth was measured and recorded every half h at 600 nm using an Infinite F200 Pro, as described previously [22]. Each analysis was performed in triplicate, and the representative growth curves were plotted.

### Glycolytic pH drop assay

The effect of NAM on *S. mutans* glycolysis was determined by modifying the method of Wang et al. [23]. *S. mutans* UA159 was harvested at the mid-logarithmic phase, washed with 0.5-mM potassium phosphate buffer containing 37.5-mM KCl and 1.25-mM MgCl_2_ (pH = 6.5), and resuspended [optical density at 600 nm (OD_600_) = 0.5] in the same solution containing sub-MIC levels of NAM (4, 8 and 16 μg/μL). The control mixture contained no NAM. Glucose was added to the mixture to achieve a final concentration of 1% (wt/vol). The decrease in pH, as a result of the glycolytic activity of the *S. mutans* UA159 cells, was monitored at 5-min intervals for 120 min (Corning pH meter 240; Corning Inc., New York, NY). The experiments were reproduced three times.

### LDH assay

*S. mutans* planktonic cells as well as biofilms were cultivated with 4, 8 and 16 μg/μL NAM for 24 h separately, with BHI or BHIS media containing no NAM serving as the control group. The crude LDH was extracted as previously described [24] and then treated with NAM at sub-MIC levels for 30 min. The activity of LDH was estimated using the LDH Activity Assay Kit (MAK066). According to the instructions of this assay kit, the absorbance at 450 nm was recorded and then calculated to quantify the enzymatic activity. The results were expressed as the percentage of ΔA450 relative to the untreated control. The experiments were performed in triplicate and reproduced three times.

### Acid tolerance assay

The effect of NAM on the acid tolerance of *S. mutans* was determined by measuring the viability of bacteria after 2 h exposure at pH = 5.0 [23,24]. *S. mutans* UA159 was grown in the TYEG medium (tryptone-yeast extract medium containing 20-mM glucose) until the cells reached the mid-logarithmic phase (OD_600_ = 0.5). Then, the cells were collected by centrifugation and resuspended (OD_600_ = 0.2) in the TYEG medium buffered with 40-mM phosphate-citrate buffer (pH = 5.0) containing sub-MIC levels of NAM (4, 8 and 16 μg/μL), and incubated at 37°C for 2 h. The control mixture contained no NAM. The samples were removed before and after incubation at pH = 5.0 for viable counts as described above. The experiments were performed in triplicate and reproduced three times.

### Colony forming units (CFUs) counting of *S. mutans* biofilms

The CFU counting method was applied to test the inhibitory activity of NAM on *S. mutans* biofilm [20,25]. As described above, the bacterial cells were diluted in fresh BHIS containing sub-MIC levels of NAM (4, 8, and 16 μg/μL) with a control mixture that contained no NAM and cultured in selected wells of a sterile 24-well microtiter plate. After incubation for 6, 12 and 24 h separately, the medium was removed, and the plate was washed with PBS three times. Adherent bacteria cells in the biofilms were resuspended in PBS and plated onto BHI agar plates after a serial dilution from 10 ^4^-fold to 10 ^6^-fold. After incubation at 37°C for 48 h, the plates were removed from the incubator to determine the CFU counts. The experiments were performed in triplicate and reproduced three times.

### Confocal laser scanning microscopy of biofilms

The *S. mutans* biofilms, cultivated on glass coverslips as described previously [26,27], were analyzed for biofilm thickness and bacterial viability using a Leica DMIRE2 confocal laser scanning microscope (CLSM) (Leica). One µM of Alexa Fluor 647-labeled dextran conjugate (Life Technologies, Grand Island, NY) was added to overnight cultures of *S. mutans* UA159 grown in BHIS. The cultures were exposed to NAM at concentrations of 0, 4, 8, and 16 μg/μL to observe its effect on biofilm formation. The plates were incubated at 37°C for 6 h under 5% CO_2_. The biofilms were stained with 2.5-μM SYTO 9 green fluorescent nucleic acid stain (480/500 nm; Molecular Probes Inc., Eugene, OR) according to the manufacturer’s instructions. The biofilm-stained samples were examined by CLSM under a × 60 oil immersion objective lens. Z sections were used to record the biofilm thickness. Vertical lines were selected randomly to analyze each image, and at least five random fields were analyzed in each experiment.

### RNA-sequencing for transcriptome analysis

*S. mutans* UA159 cells were routinely grown, diluted to 1:100, and transferred into fresh BHI broth containing 16 μg/μL NAM, and incubated at 37°C for 6 h; a medium containing no NAM was used as a control. For RNA extraction, three independent cultures of the strains with or without NAM treatment were collected and centrifuged (4,000 g, 4°C, 10 min) and treated with RNA protect (Qiagen, Valencia, CA). Total RNA was extracted, purified using RNeasy Mini kits (Qiagen), and digested with RNase-free DNase I (Qiagen). The concentration of the purified RNA samples was determined by a Nanodrop 2000 spectrophotometer (Thermo Fisher Scientific, Pittsburgh, PA). cDNA libraries were constructed from enriched mRNA samples using the Truseq^TM^ RNA sample preparation kit (Illumina, San Diego, CA). The isolation of rRNA from the total RNA was carried out using the Ribo-Zero Magnetic kit (Epicentre, WI), and the mRNA was chemically fragmented to short pieces using a 1× fragmentation solution (Ambion, MA) for 2.5 min at 94°C. Double-stranded cDNA was produced using the SuperScript Double-Stranded cDNA Synthesis kit (Invitrogen, MA). The samples were PCR-amplified for 15 cycles with paired-end primers and a randomly selected unique barcode (Illumina, San Diego, CA). RNA-sequencing libraries were constructed using the Illumina Paired End Sample Prep kit and sequenced using Illumina HiSeq 4000 [26]. Genes with a fold-change > 2.0 and a *P*-value < 0.05 were selected for further gene expression pattern discovery.

### Biocompatibility assays

The biocompatibility of NAM was assessed by the CCK-8 assay applied on human oral keratinocytes (HOKs) and human gingival epithelial cells (HGEs) following the manufacturer’s instructions (Cell Counting-Kit-8, Dojindo, Japan). The HOK and HGE cell lines were provided by the State Key Laboratory of Oral Diseases, Sichuan University. The cells were cultured in DMEM (Gibco), 100 U/mL of penicillin, and 100 mg/mL of streptomycin supplemented with 20% FBS (Gibco) in 5% CO_2_ at 37°C at a density of 5,000 cells per well in 96-well cell culture plates. The final concentrations of NAM were 4 μg/μL, 8 μg/μL, and 16 μg/μL and a medium without NAM served as negative control. After incubation under 5% CO_2_ at 37°C for 1 h, the cells were washed with sterile PBS, and 0.1 mL of the CCK-8 reagent was added to each well. The plate was then incubated at 37°C for 3 h. Then the absorbance was measured at 562 nm using a spectrometer (Power Wave XS2, Bio-Tek, VT). The statistics were normalized as cell viability, and the result of the control group was set as 100%. Each concentration was tested in three wells, and the CCK-8 assays were performed in triplicate [20].

### Rat caries model

The animal experiment was conducted with approval (approval number WCHSIRB-D-2021-156) and performed with a modified rat caries model [28,29]. Twenty-eight male specific-pathogen-free Sprague-Dawley rats aged 21 days were purchased from Dashuo Inc. (Chengdu, China). The rats were provided with ampicillin (1 g/kg) in the drinking water for 3 days, during which they were offered standard laboratory chow (Dashuo Inc). The rats were then infected orally with 0.3 mL fresh BHI medium containing *S. mutans* UA159 (OD_600_ = 0.5) for 7 days, once a day, and the infection was confirmed by oral swabbing and culturing on mitis-salivarius-bacitracin (MSB) agar plates. The infected animals were randomly assigned into four groups of seven animals: (1) 32 μg/μL NAM, (2) 16 μg/μL NAM, (3) 250 μg/mL of fluoride (as positive control), and (4) ddH_2_O (as negative control). Animals in each group were treated with the compounds above topically using a camel hair brush for 2 min twice daily for 4 weeks. The rats were fed the cariogenic Keyes 2000 diet (Trophic, Nantong, China) and 5% sucrose water ad libitum during the inoculation of *S. mutans* and the 4-week treatment [30]. The rats’ weights were recorded to monitor for signs of toxicity.

At the end of the experiment, the animals were euthanized by CO_2_ asphyxiation. The lower left jaw was aseptically dissected and sonicated in 5.0 mL of sterile saline solution (0.9%, w/v). The obtained suspensions were streaked on MSB agar plates to estimate the *S. mutans* population. The smooth and occlusal surfaces of dental caries and their severity (E, enamel only; Ds, dentin exposed; Dm, 3/4 of the dentin affected; Dx, all dentin affected) were evaluated according to Keyes’ score method [31].

### Statistical analyses

The data were analyzed using SPSS 18.0. Differences between the experimental group and the untreated control group were compared using one-way ANOVA and Tukey HSD tests. The data were considered significantly different if the *P*-value was < 0.05.

## Results

### NAM inhibited growth, acidogenicity, and acid tolerance properties of *S.* *mutans*

**Figure 1.**
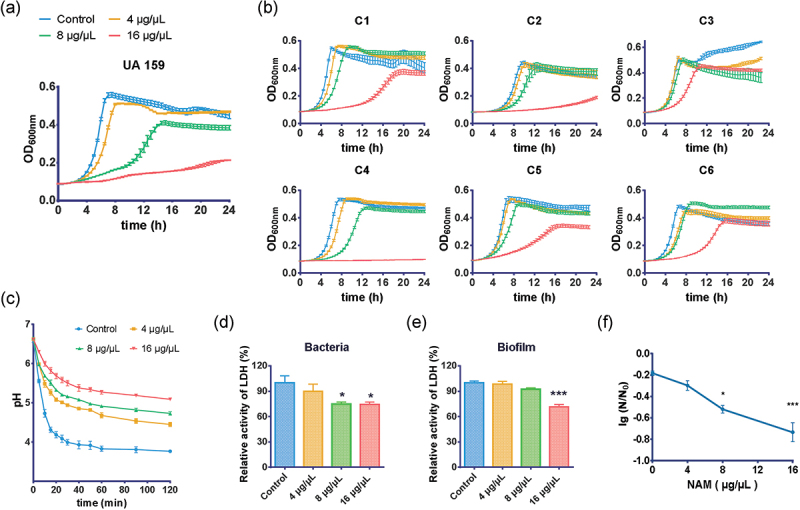
Inhibitory effect of NAM on *S. mutans* in the planktonic state. The effect of NAM on the growth of *S. mutans* UA159 (**a**) and clinical strains (**b**). The effect of NAM on the glycolytic pH drop (**c**) and lactate dehydrogenase (LDH) activity (**d** and **e**) of *S. mutans* cells and biofilm. (**f**)The effect of NAM on the survival rate of *S. mutans* at pH = 5.0. N_0_ and N represent CFU counts before and after 2 h treatment at pH = 5.0 culture, respectively. Values represent the means and standard deviations from three independent experiments (*P < 0.05, and ***P < 0.001 compared with the untreated control).

The effect of NAM on planktonic *S. mutans* growth was displayed by growth curves. The results showed the dose-dependent nature of the inhibitory effect of NAM on the proliferation of *S. mutans* planktonic cells, including UA 159 and other clinical isolates, with an extended lag phase and decreased final bacteria yield compared with the control group (Figure 1a,b).

The effects of NAM at 4, 8, and 16 μg/μL on acidogenicity were determined by monitoring the glycolytic pH drop of the *S. mutans* culture. As shown in Figure 1c, when the *S. mutans* culture was treated with NAM, its glycolytic pH decreased at a lower rate, and the terminal pH was significantly higher than the control (*P* < 0.05). In addition, the effect of NAM on *S. mutans* acidogenicity was dose-dependent.

LDH is one of the most important enzymes involved in acid production, and the LDH activity implies the *S. mutans* cariogenic potential [24]. Further assays showed that 16- and 8-μg/μL NAM suppressed the LDH activity of *S. mutans* cells (Figure 1d), and 16 μg/μL NAM significantly reduced the LDH activity of *S. mutans* biofilms (*P* < 0.001) (Figure 1e). This was consistent with the results of the glycolytic pH drop assay.

The acid resistance ability of *S. mutans* UA159 cells was also suppressed by NAM. As shown in Figure 1f, the survival rate of *S. mutans* cells after 120 min of exposure at pH = 5.0 decreased significantly in the presence of NAM compared with the untreated control. In addition, dose-dependent inhibition was observed, and NAM significantly reduced the survival rate at concentrations of 8 and 16 μg/μL (*P* < 0.05).

## NAM decreased biofilm formation and EPS synthesis of *S. mutans*

**Figure 2.**
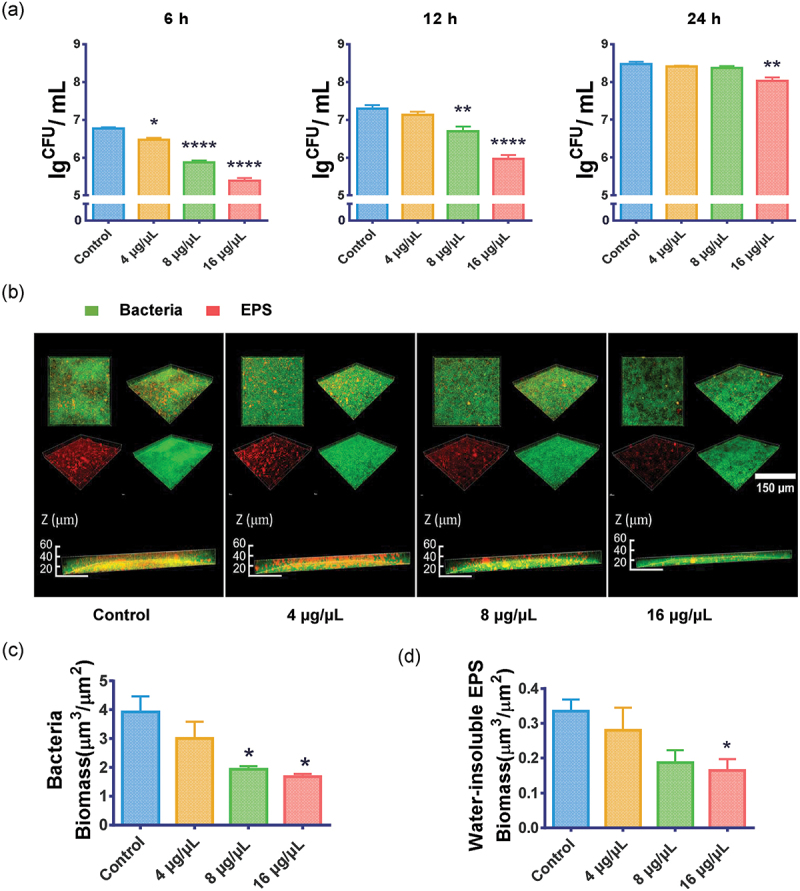
Effect of NAM on *S. mutans* biofilm. (**a**) Effect of NAM on *S. mutans* biofilm formation assessed by measuring the number of CFU in the 6 h, 12 h and 24 h biofilm. (**b**) Three-dimensional visualization and double-labeling imaging of 6 h biofilms of *S. mutans* formed on glass coverslips under NAM treatment. Live bacteria and EPS were green-labeled and red-labeled, respectively. (**c**) and (**d**) Quantification of the amounts of EPS and bacteria in each scanned layer of 6 h biofilms of *S. mutans*. The biomass of bacteria (**c**) and EPS (**d**) in each group. Values represent the means and standard deviations from three independent experiments (*P < 0.05, **P < 0.01, ****P < 0.0001 compared with the untreated control).

We tested the effect of NAM on *S. mutans* biofilm by CFU counting and CLSM observation. The CFUs in *S. mutans* biofilm were decreased when treated with NAM (Figure 2a). All of the three 6 h NAM-treated groups showed decreased CFU counts in a dose-dependent manner. In the 24 h biofilm, only 16 μg/μL NAM showed a significant decrease compared with the control group (*P< *0.05).

Figure 2b shows the representative images of 3-dimensional renderings of the *S. mutans* biofilms. In addition to thinner biofilms, both bacteria and EPS were grown more loosely and sparsely after NAM treatment. Compared with the control group, the bacterial and water-insoluble EPS biomass decreased in NAM-treated groups (Figure 2c,d, *P* < 0.05).

## Transcriptome analysis of the NAM-treated *S. mutans* strain

**Figure 3.**
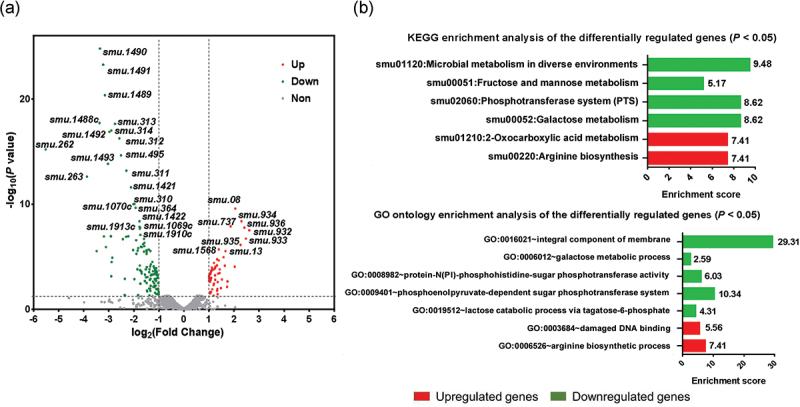
Transcriptomic analysis of NAM-treated *S. mutans* strain. (**a**) Visualization of differentially expressed genes (DEGs) with the volcano plot. (**b**) KEGG enrichment and gene ontology enrichment analysis of the DEGs using the DAVID tool (GO, gene ontology; KEGG, Kyoto Encyclopedia of Genes and Genomes).

The transcriptome analysis of *S. mutans* was carried out with or without 6 h NAM treatment to evaluate the effects of NAM on *S. mutans* gene expression. A total of 128 significantly downregulated genes and 59 upregulated genes were identified in the NAM-treated strain compared to the controls. Then gene enrichment and functional annotation clustering analyses of differentially expressed genes (DEGs) were conducted (Figure 3a, Tables S3 and S4). The expression of genes associated with carbohydrate transport, metabolism, transcription, energy production, conversion, inorganic ion transport and metabolism, nucleotide transport and metabolism, signal transduction mechanism, coenzyme transport, and metabolism were downregulated in the NAM-treated cells. By contrast, the expression of genes involved in cell cycle control, cell division, and chromosome partitioning was upregulated in the NAM-treated strain (Figure S1).

We further conducted gene annotation enrichment of DEGs using the DAVID bioinformatics tools (http://david.abcc.ncifcrf. gov/) to gain insights into the biological effects of NAM on *S. mutans*. As shown in Figure 3b, the DEGs were enriched in six pathways of the Kyoto Encyclopedia of Genes and Genomes (KEGG), with seven gene ontology (GO) terms. Notably, the integral component of membrane, sugar metabolism, the phosphotransferase system, and microbial metabolism in diverse environments were enriched with downregulated genes. In contrast, GO terms, including damaged DNA binding, the arginine biosynthesis process, and the KEGG pathways, including oxocarboxylic acid metabolism and arginine biosynthesis, were significantly enriched with upregulated genes (Figure 3b, *P*< 0.05).

**Figure 4.**
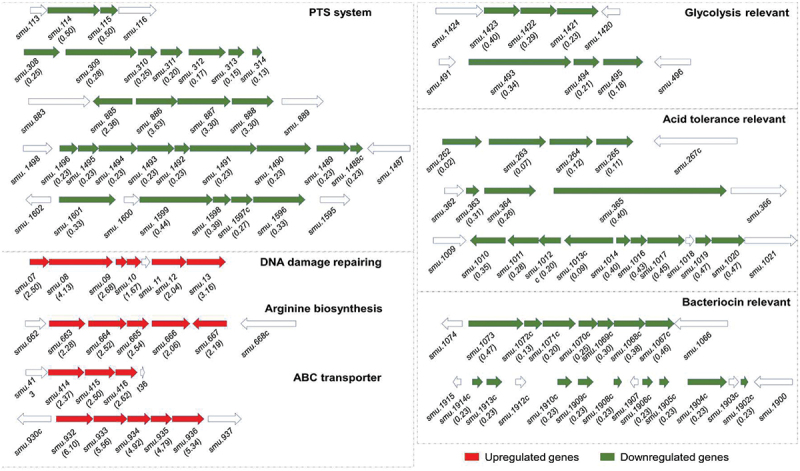
Differently expressed gene clusters. The genetic organization of differentially expressed gene clusters that were associated with *S. mutans* cariogenic virulence factors. Upregulated genes are shown in red, and downregulated genes are shown in green.

According to the *S. mutans* UA159 genome annotation obtained from the National Center for Biotechnology Information (NCBI), the downregulated gene clusters with known functions were mainly associated with the phosphotransferase system (PTS) metabolism, glycolysis and acid production, acid tolerance, and bacteriocin synthesis. In contrast, upregulated gene clusters were related to DNA damage repair and arginine synthesis (Figure 4).

## Inhibition of *S. mutans* cariogenicity in a rat caries model by NAM

**Figure 5.**
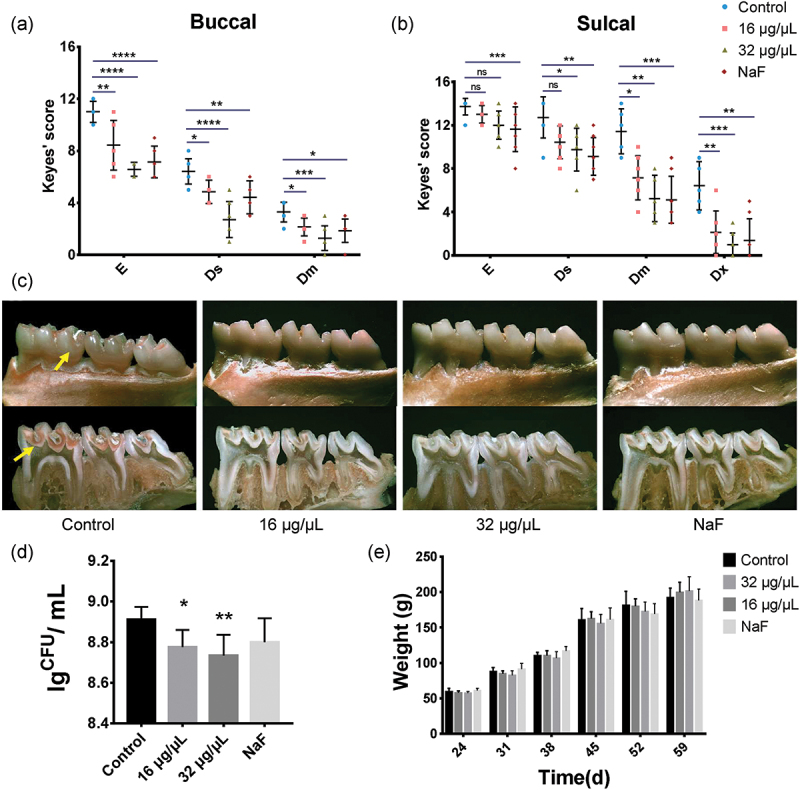
Effect of NAM on the cariogenicity of *S. mutans* in a rat caries model. (**a**) and (**b**) Keyes scores of the rats’ molar teeth on the buccal surface and sulcal surface. Each plot represents the caries score of each rat (n = 7). Error bars denote the standard deviations. E, enamel; Ds, dentin exposed, Dm, ¾ of the dentin affected, Dx, whole dentin affected. (**c**) Mandibular molars of the model rats, the upper-panel shows the buccal view, the lower-panel shows the sulcal view, and arrows indicate the representative carious lesions. (**d**) Effects of NAM on the population of *S. mutans* in rats’ dental plaque. (**e**) Effects of NAM treatments on rat weight gains. The results were averaged from three separate experiments and are presented as means ± standard deviations. *P < 0.05, **P < 0.01, ***P < 0.001, ****P < 0.0001 compared with the untreated control. ns, not statistically significant compared with the untreated control.

Since NAM was able to inhibit *S. mutans* cariogenic properties, we tested the effect of NAM on the virulence of *S. mutans* using a rat caries model. The evaluation of caries by Keyes’ scoring method revealed that NAM, as well as NaF treatment, significantly reduced the severity of carious lesions on all molar surfaces compared to that in the control group (Figure 5a-c). On smooth surfaces, NAM decreased the score from the enamel layer to the middle dentin layer while caries on extensive dentin was not detected on the buccal surface. The group treated with NAM showed significantly fewer sulcal-surface dentin lesions compared with the control group (*P*< 0.05), while a statistical difference was not found at the enamel layer. *S. mutans* CFUs recovered from the dental plaque of rats treated with NAM were decreased compared with the no-treatment group (*P*< 0.05) (Figure 5d).

In the animal experiments, all rats remained in good health with no significant differences in weight gain among the treatment groups (*P* > 0.05) during the 4-week experiment (Figure 5e).

## Discussion

The main cariogenic traits of *S. mutans* include high acidogenicity, aciduric properties, and stable biofilm formation [32]. This study showed that NAM inhibited cell growth, acid production, LDH activity, and acid tolerance, in addition to decreased biofilm formation and EPS synthesis of *S. mutans*. According to the gene expression results in the present study, gene clusters of *S. mutans* associated with the PTS systems, glycolysis, and acid tolerance responses were downregulated after NAM treatment.

In this study, a large number of genes and operons encoding glucose-specific, sorbitol-specific, galactose-specific, lactose-specific, and cellobiose-specific PTS systems were downregulated after NAM treatment (Figure 3b, Figure 4). PTS systems involve in sugar transport and metabolism, thus playing an important role in the regulation of EPS synthesis, biofilm formation, and virulence of *S. mutans*. These results suggest that NAM treatment could lead to decreased sugar uptake, further decreasing the energy and substrate sources for *S. mutans* growth and sugar metabolism [22]. These findings could also explain the delayed growth and decreased acid production exerted by NAM on *S. mutans*.

Glycolysis is the main pathway to produce acid in *S. mutans*. LDH is one of the most important enzymes in this process, and the deficiency of LDH implies that *S. mutans* lose its cariogenic potential [24]. Pyruvate is an important intermediate product during glycolysis and can be subsequently converted to end-products of fermentative metabolisms, such as lactate, acetate, and formate [33]. As the gene clusters (*smu.1421–1423* and *smu.493–495*) of glycolysis and pyruvate metabolism have been down-regulated, the acidic byproduct decreased and resulted in decreased acidogenicity (Figure 4).

The ability of *S. mutans* to quickly mount the acid tolerance responses (ATR) upon exposure to sub-lethal acidic pH is a hallmark of its cariogenicity [34]. Among all ATR, the agmatine deiminase system (AgDS) [35], which yields ammonia, CO_2_, and ATP while converting agmatine to putrescine is proposed to augment the acid resistance properties of *S. mutans* [36]. NAM exhibited an inhibitory effect on *S. mutans* acid tolerance ability and downregulated several gene clusters that are associated with encoding AgDS (*smu.262–265*) [35], and the adaptive ATR (*smu.363–365*) [37], as well as citrate metabolism (*smu.1010–1020*) [38], which increases growth yields and acid tolerance to bacteria (Figure 4).

NAM, a product of the sirtuin catalytic reaction, is a potent inhibitor of nicotinamide adenine dinucleotide (NAD^+^)-dependent lysine deacetylases. Several studies have suggested that NAM exerts its main antimicrobial effect by inhibiting sirtuin proteins. For example, NAM has antifungal activity against *C. albicans* and also effectively suppresses biofilm formation. The effect of NAM might be attributed to affecting cell wall organization and modulating the acetylation of yeast histone H3 Lys56 [19]. According to Unciti-Broceta’ study, NAM inhibited the growth and changed the morphology of *Trypanosoma brucei*, and the effects might be attributed to the inhibition of a cathepsin b-like protease [18]. Bacteria also produce sirtuins, e.g. the bacterial CobB, which is the first and major protein deacetylase identified in prokaryotes and can regulate bacterial glycolysis and growth [39,40]. *In vitro* nicotinamide inhibition of CobB has been characterized in *Escherichia coli* [41]. One possible mechanism for NAM to inhibit *S. mutans* might be through the inhibition of sirtuins proteins. Although *S. mutans* does not have a *cobB* gene, other possible deacetylates in *S. mutans* might be inhibited by NAM. Another possibility is that NAM can inhibit *S. mutans* cariogenic virulence through mechanisms, such as affecting the expression of virulence genes, as shown by the transcriptome results in this study. In addition, deacetylate inhibitors like NAM are known to induce protein acetylation of transcription factors, resulting in changes in their transcriptional activity and downstream target genes, which may also be an explanation for NAM’s effect on *S. mutans* [42]. However, further research is required to determine whether NAM would cause global changes in the protein acetylation of *S. mutans*.

The biocompatibility and *in vivo* test efficacy of antimicrobial agents is an important indicator of whether they can be widely used in clinical caries management. Therefore, we tested the cytotoxicity of NAM against human oral cells and NAM exerted minor cytotoxic effects on these cells (Figure S2). The rat caries test further supported the potential biosafety and anti-caries effect of NAM in daily and clinical use.

This study has limitations. First, the exact mechanisms of NAM still need further research. Our results showed the potential of NAM to act as an anticaries agent, but its exact efficacy should be further determined *in vivo* with optimal delivery methods like a mouth rinse, toothpaste etc. In addition, we only conducted the RNA-sequencing transcriptome analysis of planktonically grown *S. mutans*. Planktonically growing *S. mutans* and those residing in biofilms display significantly different, so the gene expression of *S. mutans* biofilms after NAM treatment remains unclear.

In conclusion, this study demonstrated the inhibitory effects of NAM on *S. mutans*’ main cariogenic properties, including growth, acidogenicity, acid tolerance, biofilm formation, and EPS synthesis. We further reported that NAM affected the expression of genes related to PTS systems, acidogenicity, and aciduricity. *In vivo* studies further suggested that NAM is an effective antimicrobial and anti-caries agent for *S. mutans*, with great potential to inhibit cariogenic biofilm and manage dental caries.
